# Studies of mutations of assembly factor Hit1 in budding yeast suggest translation defects as the molecular basis for PEHO syndrome

**DOI:** 10.1016/j.jbc.2022.102261

**Published:** 2022-07-14

**Authors:** R. Elizabeth Dreggors-Walker, Lauren N. Cohen, Sohail Khoshnevis, Virginie Marchand, Yuri Motorin, Homa Ghalei

**Affiliations:** 1 Department of Biochemistry, Emory University School of Medicine, Atlanta, Georgia, USA; 2 Graduate Program in Biochemistry, Cell and Developmental Biology (BCDB), Emory University, Atlanta, Georgia, USA; 3 Université de Lorraine, UAR2008/US40 IBSLor, CNRS-INSERM, Biopôle, Vandoeuvre-les-Nancy, France; 4 Université de Lorraine, UMR7365 IMoPA, CNRS- Biopôle, Vandoeuvre-les-Nancy, France

**Keywords:** rRNA modification, small nucleolar RNA (snoRNA), small nucleolar ribonucleoprotein (snoRNP), zinc finger HIT protein, ZNHIT3, ribosome assembly, assembly factor, ribosomopathy, PEHO syndrome, Hit1, HPG, L-homopropargylglycine, IRES, internal ribosome entry site, PEHO, progressive encephalopathy with edema, hypsarrhythmia, and optic atrophy, snoRNAs, small nucleolar RNAs, snoRNP, small nucleolar ribonucleoprotein complex, ZNHIT3, human zinc finger HIT-type containing protein 3, Zf-HIT, zinc finger HIT

## Abstract

Regulation of protein synthesis is critical for control of gene expression in all cells. Ribosomes are ribonucleoprotein machines responsible for translating cellular proteins. Defects in ribosome production, function, or regulation are detrimental to the cell and cause human diseases, such as progressive encephalopathy with edema, hypsarrhythmia, and optic atrophy (PEHO) syndrome. PEHO syndrome is a devastating neurodevelopmental disorder caused by mutations in the *ZNHIT3* gene, which encodes an evolutionarily conserved nuclear protein. The precise mechanisms by which *ZNHIT3* mutations lead to PEHO syndrome are currently unclear. Studies of the human zinc finger HIT-type containing protein 3 homolog in budding yeast (Hit1) revealed that this protein is critical for formation of small nucleolar ribonucleoprotein complexes that are required for rRNA processing and 2′-O-methylation. Here, we use budding yeast as a model system to reveal the basis for the molecular pathogenesis of PEHO syndrome. We show that missense mutations modeling those found in PEHO syndrome patients cause a decrease in steady-state Hit1 protein levels, a significant reduction of box C/D snoRNA levels, and subsequent defects in rRNA processing and altered cellular translation. Using RiboMethSeq analysis of rRNAs isolated from actively translating ribosomes, we reveal site-specific changes in the rRNA modification pattern of PEHO syndrome mutant yeast cells. Our data suggest that PEHO syndrome is a ribosomopathy and reveal potential new aspects of the molecular basis of this disease in translation dysregulation.

Ribosome biogenesis is an essential process that is tightly regulated by the action of over 200 assembly factors including proteins and noncoding RNAs (1, 2). Mutations in ribosome components or ribosome assembly factors are deleterious to the cell and can result in human diseases that are collectively termed ribosomopathies (3, 4). Among the critical ribosome biogenesis factors are small nucleolar RNAs (snoRNAs) that participate in the processing, folding, and modification of rRNAs (5, 6). snoRNAs are an abundant class of small noncoding RNAs that fall into two major classes based on their conserved elements: box H/ACA snoRNAs guide the isomerization of uridines into pseudouridines and box C/D snoRNAs are responsible for the 2′-O-methylation of rRNAs (7, 8). snoRNA-guided rRNA modifications are critical to the structure and function of the ribosome (9), and their dysregulation is linked to human diseases including cancer and devastating disorders such as dyskeratosis congenita and Treacher Collins syndrome (10, 11, 12, 13, 14, 15, 16, 17, 18, 19, 20, 21, 22).

Four evolutionarily conserved proteins, including a methyltransferase, interact with box C/D snoRNAs to form functional small nucleolar ribonucleoprotein complexes (snoRNPs) for 2′-O-methylation of over 50 nucleotides in yeast rRNAs (over 100 in humans) (6, 7). Formation of box C/D snoRNP complexes is regulated by several transiently acting assembly factors that are mostly evolutionarily conserved from yeast to humans (23). Hit1 (human zinc finger HIT-type containing protein 3, ZNHIT3), an evolutionarily conserved nuclear protein, is an assembly factor required for the biogenesis of box C/D snoRNPs (24). Both ZNHIT3 and Hit1 are members of the family of zinc finger HIT (Zf-HIT) domain-containing proteins (25, 26) and are involved in ribosome biogenesis through a critical network of protein–protein interactions required for the production of box C/D snoRNPs (24, 27). Specifically, Hit1 cooperates with another assembly factor, Rsa1 (human nuclear fragile X mental retardation protein-interacting protein 1, NUFIP1), to recruit the core box C/D snoRNP protein, Snu13 (24, 27). This is an essential early step in the biogenesis of snoRNPs which initiates the hierarchical assembly of snoRNPs (28) and prevents the catalytic activity of premature snoRNPs (27). Consistent with the critical role of Hit1, deletion of the *HIT1* gene in yeast results in low steady-state levels of box C/D snoRNAs and impairs both box C/D snoRNP assembly and ribosome biogenesis (24). Furthermore, mutations in the *ZNHIT3* gene, which encodes the human homolog of Hit1, cause a severe neurodevelopmental disorder termed PEHO syndrome (progressive encephalopathy with edema, hypsarrhythmia, and optic atrophy) (29, 30).

PEHO syndrome is a rare and fatal autosomal recessive disease characterized by progressive cerebellar atrophy, infantile spasms, arrest of psychomotor development, and a poor prognosis (31, 32, 33). ZNHIT3-associated amino acid variations that cause PEHO syndrome are found within the Zf-HIT domain of the protein (29, 30). These include the missense variation Ser31Leu (S31L) (29) and the compound heterozygous ZNHIT3 variants S31L and Cys14Phe (C14F) (30). The ZNHIT3 missense variant S31L is also found in a class of diseases with similar phenotypes to PEHO syndrome called PEHO-like syndrome (33). Mechanistically, how amino acid variations in the ZNHIT3 protein contribute to pathogenesis in PEHO syndrome remains largely unknown to date.

ZNHIT3 is required for granule neuron survival and migration in cultured mouse cells and zebrafish (29). Studies in human cell culture have shown that the ZNHIT3-S31L variation destabilizes the protein, resulting in decreased steady-state protein levels (29). Thus, loss-of-function of ZNHIT3 is suggested to cause the molecular defects observed in ZNHIT3-associated PEHO syndrome. Interestingly, however, co-immunoprecipitation assays show that the ZNHIT3-S31L variation does not compromise the interaction of the protein with its significant binding partner, NUFIP1 (29), leaving the question of how loss-of-function of ZNHIT3 causes molecular defects in PEHO syndrome.

Here, we investigate the two PEHO-causing *ZNHIT3* mutations in the model organism budding yeast to reveal the molecular basis by which they cause cellular defects and contribute to pathogenesis in PEHO syndrome. Consistent with the findings from human cell culture models, we show that introduction of the PEHO-causing mutations into yeast *HIT1* causes growth defects and leads to a decrease in steady-state levels of the Hit1 protein at higher temperatures. Using these yeast models, we reveal that as a result of Hit1 deficiency due to PEHO-linked amino acid variations, cells demonstrate a significant defect in rRNA biogenesis and a decrease in steady-state box C/D snoRNA levels. Using RiboMethSeq, we further demonstrate a site-specific reduction in the rRNA 2′-O-methylation pattern of actively translating ribosomes and a change in translation accompanied by ribosome fidelity defects. Our data offer new insights into the specific translational defects caused by loss of Hit1 and suggest that the molecular basis of PEHO syndrome pathogenesis likely lies within translation defects, adding PEHO syndrome to the list of ribosomopathies.

## Results

### PEHO syndrome mutations cause a temperature-sensitive growth defect in budding yeast

**Figure 1:**
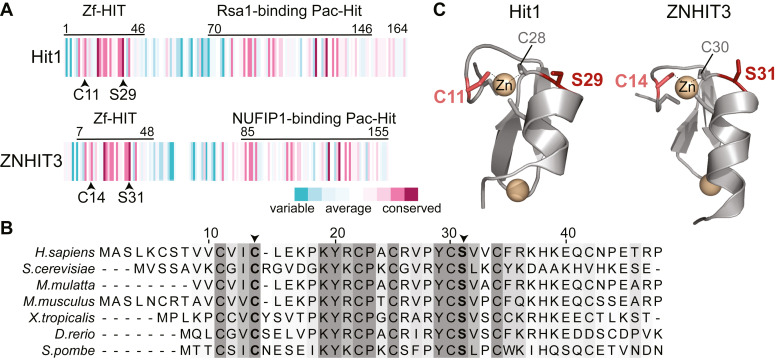
**PEHO syndrome variations in the evolutionarily conserved protein Hit1.***A*, domain organization of yeast Hit1 and human ZNHIT3 showing the conserved residues mutated in PEHO syndrome. The figure was generated using ConSurf (68). *B*, multiple sequence alignment of the Zf-Hit domains generated with Clustal Omega (69) and colored in Jalview (70). Shaded amino acids are conserved. *Darker shades* of *gray* show higher conservation. The PEHO-associated conserved cysteine and serine residues are in *bold* and marked with an *arrow*. *C*, the structure of the Zf-HIT domain of Hit1 (PDB ID: 2N95) and ZNHIT3 (PDB ID: 2YQQ) highlighting the conserved residues mutated in PEHO syndrome. PEHO, progressive encephalopathy with edema, hypsarrhythmia, and optic atrophy; Zf-HIT, zinc finger HIT; ZNHIT3, human zinc finger HIT-type containing protein 3.

The ZNHIT3 protein contains two domains that are evolutionarily conserved from yeast to human in sequence, structure, and function (24, 26, 27, 34) (Fig. 1*A*). The PEHO syndrome-causing missense mutations, which result in the ZNHIT3 variants C14F and S31L, lie within the highly conserved Zf-HIT domain of ZNHIT3 (26, 29, 30), which shares 33% sequence identity and 45% sequence similarity between human and yeast (Fig. 1*B*). Mapping the position of C14 and S31 amino acids and their yeast homologs, C11 and S29, on the available structures of the Zf-HIT domains of ZNHIT3 and Hit1 (PDB IDs 2YQQ and 2N95 (26), respectively) shows that these amino acids play identical roles in the structure of the human and yeast proteins (Fig. 1*C*). In both structures, the PEHO-linked amino acids are involved in stabilizing the protein fold by their direct (C14/C11, human/yeast) or indirect (S31/S29, human/yeast) engagement in the coordination of one of the Zn^2+^ ions within the Zf-HIT domain.

**Figure 2:**
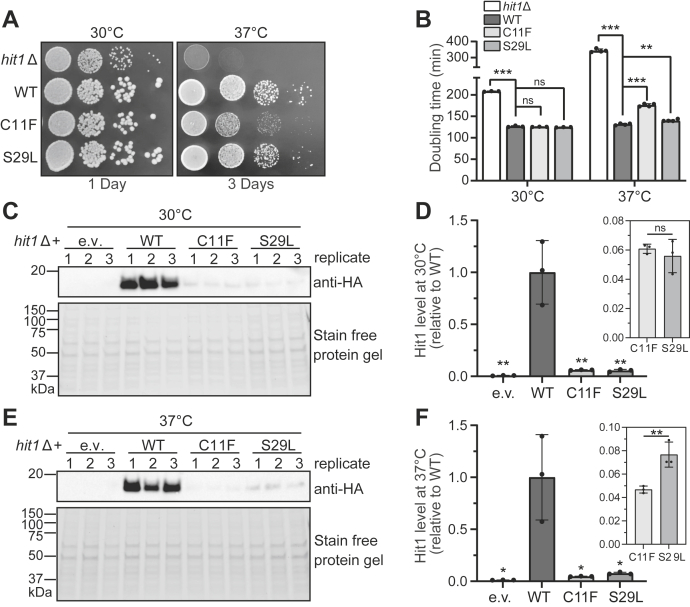
**PEHO syndrome-associated amino acid variations in Hit1 protein cause temperature-sensitive slow-growth phenotypes and result in significant loss of Hit1 protein.***A*, growth of *hit1Δ*, wildtype (WT), *hit1-C11F*, and *hit1-S29L* yeast strains on complete solid medium at 30 °C and 37 °C. *B*, doubling times of *hit1Δ*, wildtype (WT), *hit1-C11F*, and *hit1-S29L* yeast strains at 30 °C and 37 °C in a complete liquid medium. *C*, Western blot analysis of steady-state protein levels in total cell lysates of *hit1Δ* cells expressing N-terminally HA-tagged Hit1 (WT), Hit1-C11F, or Hit1-S29L at 30 °C. *D*, quantification of blots shown in panel C normalized to total protein signal relative to WT. Significance was determined using an unpaired *t* test compared to WT. Box shows a zoomed-in view of the relative steady-state protein levels in *hit1-C11F* and -*S29L* cells. *E*, Western blot analysis of steady-state protein levels in total cell lysates of *hit1Δ* cells expressing N-terminally HA-tagged Hit1 (WT), Hit1-C11F, or Hit1-S29L at 37 °C. *F*, quantification of blots shown in panel C normalized to total protein signal relative to WT. Significance was determined using an unpaired *t* test compared to WT. *Box* shows a zoomed-in view of the relative steady-state protein levels in *hit1-C11F* and -*S29L* cells. For all graphs, bars represent the mean and standard deviation of three biological replicates. ns: not significant; ∗*p* < 0.05; ∗∗*p* < 0.01; ∗∗∗*p* < 0.001. PEHO, progressive encephalopathy with edema, hypsarrhythmia, and optic atrophy.

Previous work has established the functional conservation of Hit1 between eukaryotes, particularly between yeast and human (24, 34, 35). To gain insight into the molecular basis of defects caused by PEHO-associated Hit1 mutations, we used CRISPR-Cas9 genome editing to generate budding yeast models that encode the C11F or S29L variants of the *HIT1* gene. Using growth assays on solid medium, we first assessed the growth of *hit1-C11F* and *hit1-S29L* yeast strains relative to wildtype control cells. Our results show that while neither mutant strain has a growth defect at 30 °C, yeast expressing C11F grows significantly slower than wildtype control cells at 37 °C (Fig. 2*A*). Furthermore, the yeast strain expressing the Hit1-S29L variant has smaller colony size compared to the wildtype. To quantify and confirm our findings, we next measured the growth of cells expressing Hit1 variants in liquid medium at 30 °C and 37 °C. Similar to growth on plates, both *hit1-C11F* and *hit1-S29L* cells show a growth defect at 37 °C in liquid medium, with that of C11F being more severe than S29L. Notably, the growth defect for both mutants is less severe than the defect observed upon deletion of *HIT1* (Fig. 2*B*). These results indicate that PEHO-linked Hit1 variants cause cellular defects in yeast models, providing a convenient model system to further explore the molecular defects caused by these mutations.

### PEHO syndrome mutations in budding yeast result in decreased steady-state Hit1 protein levels

Because both C11F and S29L mutants show a slow-growth phenotype at 37 °C, similar to the temperature-sensitive phenotype reported for *hit1Δ* cells, we next analyzed the steady-state Hit1 protein levels in wildtype control and *hit1* mutant cells at 30 °C and 37 °C. For this analysis, we transformed *hit1Δ* cells with plasmids that constitutively express the triple-HA (3×HA)-tagged or untagged wildtype or variant Hit1 (C11F or S29L). To test the effect from addition of the HA tag, we first compared the growth of HA-tagged and untagged *hit1* mutants relative to the wildtype control. This analysis confirmed that cells expressing HA-tagged wildtype Hit1 grow similar to those expressing untagged wildtype Hit1, and the untagged *hit1* mutant strains have the same slow-growth phenotype as the HA-tagged *hit1* mutants (Fig. S1). We then analyzed the steady-state levels of the 3×HA-tagged Hit1 protein by Western blotting of total cell protein lysates. Both *hit1-C11F* and *hit1-S29L* mutants show significantly reduced steady-state Hit1 protein levels at 30 °C (Fig. 2, *C–D*) and 37 °C (Fig. 2, *E–F*). These data show a significant reduction of steady-state Hit1 PEHO variant levels and are in line with previous observations regarding a decrease in ZNHIT3 protein levels for the S31L PEHO variant in HeLa cells (29).

### PEHO syndrome mutations in budding yeast lead to rRNA processing defects and decreased steady-state box C/D snoRNA levels

**Figure 3:**
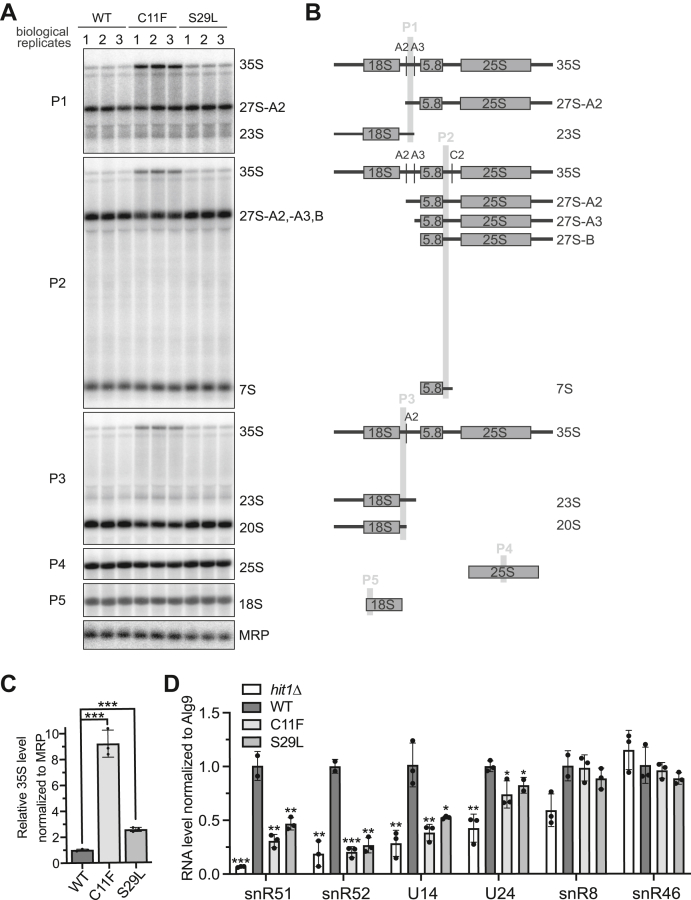
**PEHO syndrome mutations result in defective rRNA processing and a reduction of steady-state box C/D snoRNA levels.***A*, Northern blot analysis of steady-state levels of precursor rRNA levels in wildtype control (WT), *hit1-C11F*, and *hit1-S29L* yeast grown at 37 °C. Each lane represents an independent biological replicate. *B*, schematic of the yeast precursor rRNAs and the binding sites of probes used in panel A (P1-P5). *C*, quantification of the blots shown in panel A. 35S signal for each biological replicate was normalized to MRP signal. Quantifications are shown relative to wildtype control (WT). *D*, RT-qPCR quantification of steady-state snoRNA levels in *hit1Δ*, wildtype control (WT), *hit1-C11F*, and *hit1-S29L* yeast grown at 37 °C. The levels of four box C/D snoRNAs and two H/ACA snoRNAs (controls) are shown. For all graphs, bars represent the mean and SD of 2 to 4 biological replicates. Significance was determined using an unpaired *t* test compared to WT. n.s.: ∗*p* < 0.05; ∗∗*p* < 0.01; ∗∗∗*p* < 0.001. PEHO, progressive encephalopathy with edema, hypsarrhythmia, and optic atrophy; snoRNAs, small nucleolar RNAs.

Loss of Hit1 protein results in ribosome biogenesis defects (24). We therefore tested whether impaired ribosome biogenesis contributes to the growth defect of the PEHO syndrome Hit1 mutants, which have decreased steady-state Hit1 protein levels. To this end, we extracted total cell RNA from wildtype control and mutant C11F or S29L yeast cells grown at 37 °C and analyzed rRNA processing by Northern blotting (Fig. 3*A*). In budding yeast, mature rRNAs are processed from a long polycistronic 35S precursor rRNA (Fig. 3*B*) (2, 36). rRNA processing in wildtype yeast typically occurs cotranscriptionally such that 35S levels are not detectable (2, 37, 38). We observe a striking increase in the levels of 35S precursor rRNA in the *hit1-C11F* cells. Similarly, the 35S levels slightly but significantly increase in the *hit1-S29L* mutant (Fig. 3, *A* and *C*). These data suggest an early impairment of the cotranscriptional step of rRNA processing in PEHO-mutant yeast strains, indicating a potential impact from the Hit1 variants on the quantity of mature ribosomes and cellular translation.

Hit1 is a box C/D snoRNP assembly factor necessary for the maintenance of box C/D snoRNA levels that are required for proper rRNA processing (24). We therefore tested the extent to which the loss of Hit1 protein in PEHO-mutant yeast cells would decrease box C/D snoRNA levels. We used RT-qPCR to quantify the snoRNA levels of both *hit1* variants compared to wildtype control and *hit1Δ* cells at 37 °C (Fig. 3*D*). Box C/D snoRNA levels were compared to the Alg9 mRNA and box H/ACA snoRNAs as controls, as these RNAs are not regulated by Hit1. Both PEHO-linked Hit1 mutants show an intermediate, but significant, loss of box C/D snoRNAs relative to wildtype control and *hit1Δ* cells. This loss is specific to box C/D snoRNAs since box H/ACA snoRNA levels are not significantly changed relative to wildtype. Of the two PEHO syndrome mutants, *hit1-C11F* shows the more severe loss of box C/D snoRNAs (Fig. 3*D*). These data are consistent with the observation that *hit1-C11F* cells have more severe growth and rRNA processing defects and show a greater loss of Hit1 protein levels.

### PEHO syndrome mutations cause translation defects, impact the fidelity of protein synthesis, and result in changes in sensitivity of yeast cells to translation inhibitors

**Figure 4:**
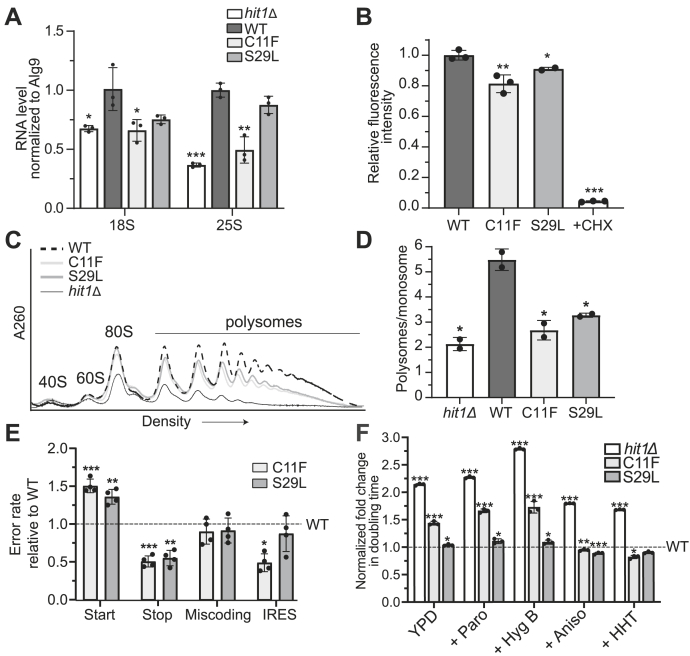
**PEHO syndrome mutations cause translation defects.***A*, RT-qPCR quantification of steady-state mature rRNA levels in *hit1Δ*, wildtype control (WT), *hit1-C11F*, and *hit1-S29L* yeast grown at 37 °C. *B*, quantification of nascent protein synthesis by Click-iT HPG in wildtype control and *hit1-C11F* cells. *C*, polysome profiles of *hit1Δ* yeast cells expressing wildtype HA-Hit1, HA-Hit1-C11F, HA-Hit1-S29L, or an empty plasmid grown at 37 °C. Clarified cell extracts were resolved on a sucrose gradient and scanned at 260 nm. *D*, the ratios of polysomes to monosomes (P/M) determined by polysome profiling and area under the curve analysis of two biological replicates are indicated. *E*, expression of Firefly and Renilla luciferase was measured in wildtype, *hit1-C11F*, or *hit1-S29L* yeast harboring dual-luciferase plasmids (listed in Table S3). The ratio of Firefly luciferase to Renilla luciferase is shown normalized to their respective control plasmids and relative to WT. *F*, doubling times of *hit1Δ*, *hit1-C11F*, and *hit1-S29L* yeast in liquid media in the presence of translation inhibitors relative to wildtype control cells (WT): Paromomycin (Paro), hygromycin B (Hyg B), anisomycin (Aniso), and homoharringtonine (HHT). The fold change is calculated by dividing the doubling time of *hit1Δ, hit1-C11F*, or *hit1-S29L* cells by the mean wildtype doubling time in each condition. Bars represent the mean and SD of 2 to 4 biological replicates. Significance was determined relative to wildtype using an unpaired *t* test. ∗*p* < 0.05; ∗∗*p* < 0.01; ∗∗∗*p* < 0.001. PEHO, progressive encephalopathy with edema, hypsarrhythmia, and optic atrophy.

Ribosome biogenesis defects can lower cellular ribosome concentration and cause defects in translation (39, 40). Similarly, loss of snoRNAs and/or rRNA modifications can cause translation fidelity defects and impact the ability of ribosomes to initiate mRNA translation from internal ribosome entry sites (IRESs) (6, 9, 41, 42, 43, 44, 45). To test whether translation is impacted by PEHO syndrome mutations in yeast, we first investigated the steady-state levels of the mature 18S and 25S rRNAs in the PEHO syndrome mutants by RT-qPCR. At 37 °C, both *hit1Δ* and *hit1-C11F* cells have reduced 18S and 25S rRNA levels relative to wildtype. Cells expressing *hit1-S29L* exhibit the same trend, although the reduction is not significant compared to wildtype (Fig. 4*A*).

To measure how translational output is affected in PEHO syndrome *hit1* mutant strains, we next performed an L-homopropargylglycine (HPG) incorporation assay to quantify global translation in bulk at a given time point. HPG is a methionine analog with an alkyne moiety, which can be incorporated into newly synthesized proteins, allowing their detection by a click reaction with Alexa Fluor azide. We compared the incorporation of HPG into the newly synthesized proteins 30 min after the addition of HPG to the media. These results show that in both *hit1-C11F* and *hit1-S29L* cells, the translational output is significantly decreased relative to wildtype control cells (Fig. 4*B*). We further examined changes in bulk translation of PEHO mutant yeast by examining the effects on total polysome profiles. Cells were grown to mid-log phase and treated with cycloheximide before harvesting to stall polysomes. Free RNA-containing complexes were then resolved from small (40S) and large (60S) ribosomal subunits, monosomes, and polysomes by sedimentation through sucrose density gradients. Both the *hit1-C11F* and *hit1-S29L* mutations reduce the ratio of polysomes to monosomes, indicating a reduction in bulk translation compared to wildtype control cells (Fig. 4, *C* and *D*).

To reveal the nature of translational defects in *hit1-C11F* mutant cells, we used a series of previously established reporter plasmids to check the fidelity of protein synthesis (46, 47, 48, 49). In these reporter plasmids, the translation of Firefly luciferase depends on alternate start codon selection, stop codon readthrough, miscoding of the CGC (Arg) codon by tRNA^His^GUG, or initiation from an IRES element, while the translation of Renilla luciferase is constitutive and is used as an internal control. Relative to wildtype control cells, both PEHO syndrome mutants show a significant reduction in stop codon readthrough, a significant increase in initiation from a near-cognate UUG start codon, but no significant change in miscoding (Fig. 4*E*). We also detect a significant decrease in IRES recognition in *hit1-C11F* cells relative to wildtype control, which is not observed for the *hit1-S29L* cells (Fig. 4*E*). Together, these data establish that PEHO syndrome-associated Hit1 variants cause translation defects and impair the fidelity of protein synthesis in yeast models. Given the evolutionarily conserved function of Hit1 and ZNHIT3 and the conservation of the ribosome biogenesis and translation pathways between yeast and human, these data strongly suggest that translation can be both globally and/or specifically affected in PEHO syndrome.

To gain further insight into the cause of translation defects observed in *hit1* mutant yeast cells, we tested whether these defects could be a result of ribosome structural changes. For this purpose, we used translation inhibitors that specifically bind to the small or large ribosomal subunits as tools for assessing changes in ribosome structure. Paromomycin and hygromycin B were used to probe defects near the decoding center, whereas anisomycin and homoharringtonine were chosen to probe changes near the peptidyl transferase center (50). Compared to wildtype cells, both *hit1Δ* and *hit1-C11F* cells are slightly more sensitive to the addition of paromomycin and hygromycin B, aminoglycosides that specifically bind to the small ribosomal subunit at the decoding center. In the presence of these drugs, the fold change in doubling time between wildtype and *hit1-C11F* strains is approximately 1.7-fold, compared to that with no drug at 1.4-fold. Similarly, in the presence of these drugs, the fold change between *hit1Δ* and wildtype increases from 2.1-fold to 2.3- to 2.8-fold (Fig. 4*F*). Cells expressing Hit1-S29L exhibit minimal changes in sensitivity to paromomycin and hygromycin B. Interestingly, *hit1Δ*, *hit1-C11F*, and *hit1-S29L* cells are less sensitive to anisomycin and homoharringtonine, inhibitors that bind in the large ribosomal subunit tRNA A-site. In the presence of anisomycin and homoharringtonine, the fold change in doubling time between wildtype and both *hit1* mutants drops to 0.8- to 0.9-fold. Similarly, the fold change between wildtype and *hit1Δ* cells drops from 2.1-fold to 1.7- to 1.8-fold (Fig. 4*F*). Overall, cells lacking Hit1 appear to follow the same trend of drug sensitivity as those expressing Hit1-C11F or Hit1-S29L, suggesting that the growth phenotypes observed in *hit1* mutant strains in response to translation inhibitors are a consequence of low steady-state Hit1 protein levels. Together, these data indicate that there may be changes in both the small and large ribosomal subunits that can affect the binding between the ribosome and ligands such as translation inhibitors, tRNAs, or IRES elements.

### The rRNA 2′-O-methylation pattern of the *hit1-C11F* mutant is altered

**Figure 5:**
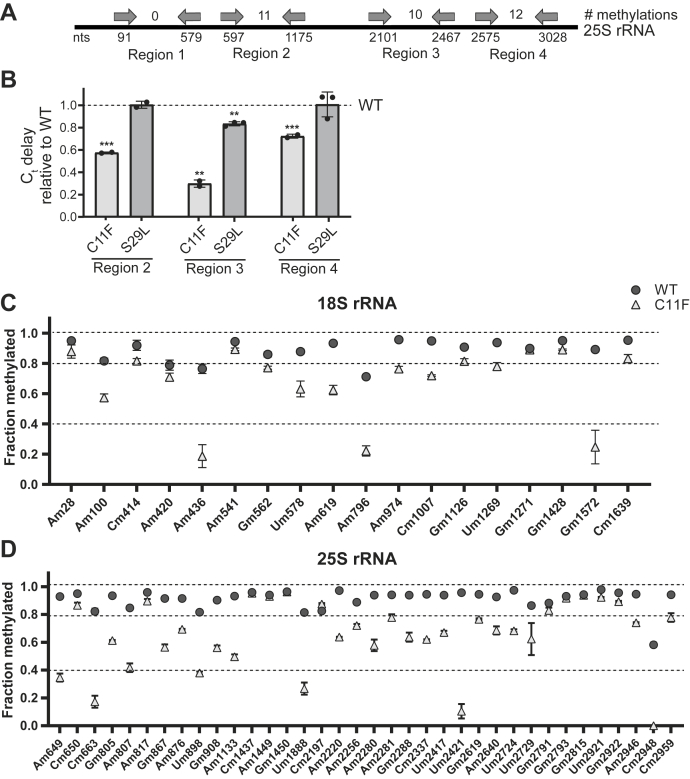
**The *hit1-C11F* mutation results in site-specific reductions of 2′-O-methylation levels of polysomal rRNAs.***A*, schematic of regions of 25S probed in *B*. *B*, total RNA from wildtype control, *hit1-C11F*, and *hit1-S29L* cells were extracted, and three regions of 25S rRNA (regions 2–4) were probed for their 2′-O-methylation levels by reverse transcription at *low* dNTP concentration followed by qPCR in biological triplicate. Region 1 was used for normalization. Bars represent the mean and SD of 2 to 3 biological replicates. Significance was determined relative to wildtype using an unpaired *t* test. ∗∗*p* < 0.01; ∗∗∗*p* < 0.001. *C* and *D*, RiboMethSeq analysis of polysomal RNA extracted from wildtype control and *hit1-C11F* cells grown at 37 °C. MethScores of 18S rRNA (*C*) and 25S rRNA 2′-O-methylated nucleotides (*D*). Data indicate the mean and SD of three biological replicates.

Because box C/D snoRNA levels are decreased in both PEHO syndrome mutants and we observed global and distinct translational defects, we hypothesized that PEHO syndrome mutations may alter the rRNA 2′-O-methylation pattern. To assess the level of rRNA 2′-O-methylation in PEHO mutant yeast cells, we first employed a reverse transcription-based assay to globally analyze the 2′-O-methylation of the 25S rRNA in total RNA that was isolated from wildtype control, *hit1-C11F* or *hit1-S29L* cells. We performed this analysis on three regions of the 25S rRNA which have 11, 10, or 12 2′-O-methylations, respectively, and used a region without any rRNA 2′-O-methylations as an internal control for normalization (Fig. 5*A*). The results from this assay show a significant hypo 2′-O-methylation in all three regions of rRNA isolated from the *hit1-C11F* mutant yeast (Fig. 5*B*). However, we only observe a slight hypo 2′-O-methylation in one region of 25S rRNA for the *hit1-S29L* mutant cells. Specifically, in region 2 of the 25S rRNA, the *hit1-C11F* cells show ∼70% reduction in the level of rRNA 2′-O-methylation, compared to a modest 20% reduction in the *hit1-S29L* mutant cells (Fig. 5*B*). These data are in line with the less severe growth phenotype and translational defects we observe in cells expressing Hit1-S29L compared to those expressing Hit1-C11F (Figs. 2*A* and 4, *A*–*E*). The data also provide an explanation for the observed decrease in the ability of ribosomes from *hit1-C11F* cells to initiate translation from an IRES, as observed for other cells that have rRNA hypo 2′-O-methylation (9, 15, 41).

**Figure 6:**
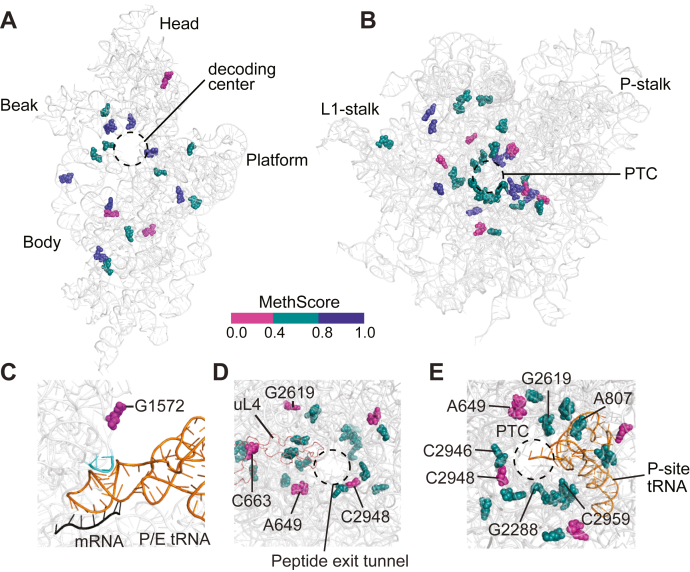
**The *hit1-C11F* mutant impacts 2′-O-methylation of nucleotides at key ribosome positions.***A*, the position of each rRNA 2′-O-methylation site is marked on the structure of 18S rRNA (PDB ID: 6GQV). *B*, the position of each rRNA 2′-O-methylation site is marked on the structure of 25S rRNA (PDB ID: 6GQV). In panels A and B, MethScores > 0.8 indicate stably methylated sites and are shown in the *dark blue*; MethScores between 0.4 and 0.8 are considered variable sites and shown in *teal green*; MethScores below 0.4 indicate hypo 2′-O-methylated sites and are in magenta. *C*, view of the small ribosomal subunit showing variable and hypo 2′-O-methylated sites near the decoding center. The critical and conserved ridge (nucleotides 1575–1578), which forms a steric block between the P- and E-site tRNA, is located near the hypo 2′-O-methylated site G1572 and is shown in *light blue*. tRNA is colored in *orange* and mRNA in *black* (PDB ID: 3j78). Views of peptide exit tunnel (*D*) and peptidyl transferase center (*E*) show numerous variable and hypo 2′-O-methylated sites located at or near these functionally important regions.

To reveal how the rRNA 2′-O-methylation pattern is affected in yeast expressing Hit1-C11F on both the small and large ribosomal subunits, we subjected the polysomal RNA isolated from actively growing wildtype control and *hit1-C11F* mutant cells to RiboMethSeq analysis (51). Our results show that most rRNA sites in polysomal RNA of wildtype control cells are fully methylated when cells are grown at 37 °C (Fig. 5, *C* and *D*) (51). However, rRNAs isolated from polysomes of *hit1-C11F* cells show significant global hypo 2′-O-methylation, with different rRNA locations being affected to different extents. Specifically, in *hit1-C11F* cells, ∼44% (8/18) of 2′-O-methylation sites on the 18S rRNA are variable (MethScore 0.4–0.8) and ∼17% (3/18) are hypo 2′-O-methylated (MethScore < 0.4) (Fig. 5*C*). On the 25S rRNA, ∼53% of 2′-O-methylation sites are variable (19/36), and ∼17% of sites (6/36) are hypo 2′-O-methylated (Fig. 5*D*). These data indicate that actively translating ribosomes from *hit1-C11F* cells are hypo 2′-O-methylated and have a distinct pattern of rRNA modification compared to those in wildtype control cells. The three most hypo 2′-O-methylated sites on the 18S rRNA are located in the head and body of the small ribosomal subunit (Fig. 6*A*). The hypo 2′-O-methylated position 18S-A436 is located near the binding site of eIF5B and eEF2 and in vicinity of uS12 (Fig. S3, *A* and *B*). Similarly, the hypo 2′-O-methylated nucleotide 18S-G1572 is located near functional ribosomal sites close to the eIF2A binding site and in vicinity of the P-tRNA binding site on the small ribosomal subunit (Figs. 6*C* and S3*C*). On the large ribosomal subunit, the hypo 2′-O-methylated sites are largely clustered around the peptidyl transferase center and the peptide exit tunnel (Fig. 6, *B*, *D*, and *E*). In summary, the RiboMethSeq analysis reveals that the PEHO-associated Hit1-C11F variant affects the rRNA 2′-O-methylation of actively translating ribosomes in a site-specific manner including at functionally important sites on both ribosomal subunits.

## Discussion

In this study, using budding yeast as a model organism, we reveal the molecular defects caused by the ZNHIT3 pathogenic missense mutations which cause PEHO syndrome. We analyze both reported PEHO syndrome-causing ZNHIT3 variants by introducing them into the *HIT1* gene in yeast (*hit1*-*C11F* and -*S29L*) and assess their impact on yeast cell growth, ribosome biogenesis, and cellular translation. While both amino acid variants destabilize the steady-state Hit1 protein levels, the Hit1-C11F variant causes more severe growth defects, lower box C/D snoRNA levels, and more prominent defects in rRNA processing compared to Hit1-S29L (Figs. 2 and 3). The majority of the studied ZNHIT3-associated PEHO syndrome cases to date are caused by homozygous expression of the ZNHIT3-S31L variant (yeast Hit1-S29L) (33). PEHO syndrome can also be caused by the compound heterozygous variants C14F and S31L (30). Because the phenotype of yeast cells expressing the Hit1-C11F variant is more severe than that of the Hit1-S29L variant, we anticipate that homozygous ZNHIT3-C14F variants may be incompatible with survival and thus underrepresented in patients.

Our data show that low steady-state Hit1 protein levels cause significant rRNA processing impairments and result in lower steady-state levels of both the modifying and the processing box C/D snoRNAs (Fig. 3). The observed rRNA processing defects in *hit1* mutant cells are reminiscent of defects observed for other early ribosome biogenesis factors as expected for a snoRNP assembly factor (52, 53, 54). Given the conserved role of Hit1 and ZNHIT3 between yeast and human (24, 27), it is likely that the ZNHIT3 variants also cause ribosome biogenesis defects in human cells. Future studies are required to reveal the effect of low steady-state levels of PEHO-causing ZNHIT3 protein variants (29) on rRNA processing in human cells.

Deletion of Hit1 leads to decreased box C/D snoRNA levels and impaired rRNA processing (24). However, the specific outcomes of these changes for cellular translation were unexplored to date. The finding that Hit1-C11F expression results in heterogeneous modification of rRNA in polysomal fractions, representing actively translating ribosomes, strongly suggests the existence of distinct pools of translating ribosomes in the mutant cells (Fig. 4). Strikingly, even though the levels of box C/D snoRNAs and their corresponding 2′-O-methylations are generally decreased, Hit1 loss preferentially affects 2′-O-methylation of certain rRNA positions more than others. The pattern of hypo 2′-O-methylation observed in Hit1 mutant cells resembles changes recently identified for a mutant of another box C/D snoRNP assembly factor, Bcd1 (9). This similarity suggests that certain rRNA 2′-O-methylation sites are more vulnerable than others when snoRNP assembly is impaired. Interestingly, many of the vulnerable sites are conserved between yeast and humans. These include one of the three hypo 2′-O-methylated positions in the 18S (A436) and four of the hypo 2′-O-methylated sites in the 25S rRNA (A649, C663, U1888, U2421). Future studies are required to address whether PEHO-causing mutations in ZNHIT3 affect the 2′-O-methylation pattern of human ribosomes similarly.

**Figure 7:**
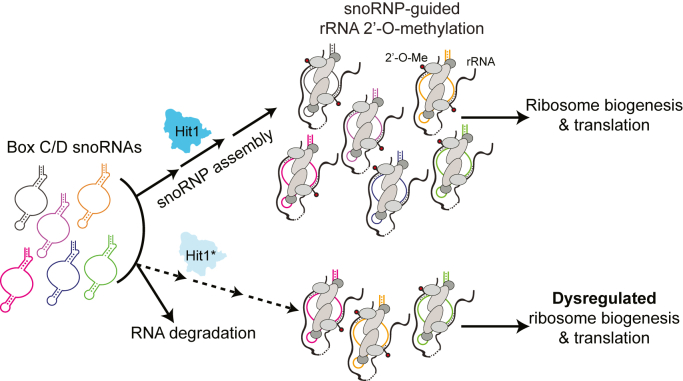
**Model of the molecular mechanism of cellular defects caused by PEHO syndrome-associated Hit1 variants.** Hit1 is an assembly factor critical for the formation of box C/D snoRNPs. The steady-state levels of Hit1 regulate the cellular box C/D snoRNA levels, the accuracy of rRNA 2′-O-methylation pattern, ribosome biogenesis, and translation. PEHO-linked *hit1* mutant yeast cells have lower steady-state Hit1 levels that result in lower levels of box C/D snoRNAs and negatively impact snoRNP assembly, which leads to impaired ribosome biogenesis and rRNA 2′-O-methylation, ultimately altering the cellular translational program. PEHO, progressive encephalopathy with edema, hypsarrhythmia, and optic atrophy; snoRNA, small nucleolar RNA; snoRNP, small nucleolar ribonucleoprotein complex.

Loss of box C/D snoRNAs in *hit1-C11F* cells does not correlate well with the decrease in their corresponding 2′-O-methylations. For example, in the *hit1-C11F* mutant yeast, the steady-state levels of snR52 are decreased to 20% compared to the wildtype control (Fig. 3*D*), while the 2′-O-methylation levels at sites guided by snR52 (18S-A420 and 25S-U2921) are stable relative to wildtype (relative MethScores around 0.9, Fig. 5). These data are in line with previous studies that yeast and human rRNA 2′-O-methylation levels do not correlate well with the level of their corresponding box C/D snoRNAs (9, 55). Our data also strongly suggest that while a threshold level of each snoRNA is sufficient to guide 2′-O-methylation of the majority of rRNAs (9), differential stability, and/or efficiency of snoRNP assembly of each snoRNA impacts the active pool of available snoRNAs, the pattern of rRNA modification, ribosome biogenesis, and translation when snoRNP biogenesis is defective (Fig. 7).

Several studies have reported heterogeneity in the rRNA 2′-O-methylation pattern in both yeast and human cells (15, 21, 41, 51, 55, 56, 57, 58, 59). However, it was unclear whether globally hypo 2′-O-methylated rRNAs assembled into functional ribosomes or if a population of hypo 2′-O-methylated rRNAs comprised immature or inactive ribosomes, while a more methylated population of rRNAs comprised translationally active ribosomes. Here, we provide a global quantification of polysomal rRNA 2′-O-methylations using RiboMethSeq. Our results show that significantly hypo 2′-O-methylated rRNAs are part of translating ribosomes. Furthermore, similar to the previous report on the 2′-O-methylated 18S rRNA site A100 (57), our data indicate that rRNA 2′-O-methylation levels quantified in the total RNA pool are not significantly different than that of actively translating ribosomes (Fig. S2). This suggests that quantification of rRNA 2′-O-methylation from the total RNA pool is an appropriate proxy for assessing the methylation status of actively translating ribosomes.

Several important conclusions can be drawn from this study that likely have significant implications for understanding the molecular basis of diseases that arise from defects in snoRNP biogenesis. Our data reveal that defects in snoRNP assembly can cause distinct translational defects. In both PEHO syndrome mutant yeast strains, we observe a decrease in global translation, an increase in initiation from a near-cognate start codon, and hyperaccuracy in stop codon readthrough. Both lower translation fidelity and higher accuracy in translation can negatively affect cellular homeostasis and growth and cause detrimental cellular defects (60). The trend of decreased translation paired with decreased stop codon readthrough is also observed in other yeast mutants lacking rRNA modifications (43). For example, the lack of 2′-O-methylation at the 25S-A2256 site in h69 leads to decreased stop codon readthrough (43). In *hit1-C11F* cells, A2256 is variably methylated (MethScore 0.7); however, loss of multiple adjacent modifications can also have a combinatorial effect on translation fidelity (43). Loss of rRNA modifications in the decoding center of bacterial ribosome leads to both increased alternate start site usage and decreased stop codon readthrough (61), the same combination of fidelity defects we observe in the PEHO syndrome yeast mutant cells. While we do not observe a significant change in the modification of sites at the decoding center, PEHO syndrome mutant yeast cells are sensitive to translation inhibitors which bind at the decoding center (Fig. 4*F*). It is possible that hypo 2′-O-methylations in *hit1-C11F* ribosomes at sites neighboring the decoding center impact the events at the decoding center *via* long-range effects (9). For example, the hypo 2′-O-methylated site 18S-G1572 is a few nucleotides away from residues that provide a steric block for the P-site tRNA (Fig. 6*C*). Impairments in start codon selection and stop codon readthrough are also observed in other ribosomopathy models. For example, ribosomopathy-causing mutations in uS12, positioned near the hypo 2′-O-methylated nucleotide 18S-A436 (Fig. S3*B*), result in increased non-AUG translation initiation in bacteria (62). Additionally, a yeast variant of Dkc1 that is linked to the dyskeratosis congenita and causes a global decrease in rRNA pseudouridylation levels leads to lower rates of stop codon readthrough (63). On the 25S rRNA, many of the vulnerable sites are located around the peptidyl transferase center (Fig. 6*E*), where we observe a loss of sensitivity to translational inhibitors in both mutants. The translation defects observed in PEHO syndrome mutant yeast cells could arise from changes in ribosome concentration or assembly defects that are caused by insufficient amounts of box C/D snoRNAs or lack of their corresponding modifications. Future studies are required to address the contribution of each of these factors to the observed defects and determine the effect from the local or long-range impact of rRNA modification changes on ribosomal protein, translation factor, or ligand binding.

PEHO syndrome is a neurodevelopmental disease characterized by the loss of cerebellar granule neurons. The tissue-specific defects of PEHO syndrome may arise from translation changes that impact cellular differentiation and neurodevelopment. Ribosome profiling performed in a human cell line reveals changes throughout neuronal differentiation in upstream ORF translation associated with near-cognate start codon usage which could impact neuronal differentiation (64). Furthermore, 2′-O-methylation has been implicated in differentiation and development in human cell lines and zebrafish models (21, 65). We, therefore, propose that changes in the 2′-O-methylation pattern, ribosome number, or the fidelity of translation in PEHO syndrome may alter the translation of specific mRNAs involved in neuronal differentiation to cause tissue-specific defects. Together, our results offer novel insights into molecular mechanisms of cellular defects caused by snoRNP assembly impairment and suggest that PEHO syndrome is likely a ribosomopathy arising from defective ribosome biogenesis due to loss of box C/D snoRNAs and change of the rRNA 2′-O-methylation pattern.

## Experimental procedures

### Yeast strains and CRISPR-Cas9 genome editing of Hit1

All yeast strains used in this study are listed in Table S1, oligonucleotides are in Table S2, and plasmids are in Table S3. Genome editing was carried out as described previously (66). In brief, a primer was designed for mutagenesis of the guide recognition sequence and used to amplify the pCAS9 vector (Addgene 60,847) (66). A double-stranded 160-mer repair DNA was generated, and the BY4741 yeast cells were transformed with pCAS9-Hit1 and the PCR product. Transformants were plated onto YPD plates supplemented with G418 and allowed to grow for 72 h at 37 °C. Individual colonies were selected, and mutations in each colony were confirmed by sequencing and further validated in growth assays after transforming each strain with a plasmid expressing the wildtype control *HIT1* gene.

### Yeast growth assays

For growth curves, BY7471, *hit1-C11F*, *hit1-S29L*, or *hit1*Δ cells were grown in YPD to mid-log phase (*A*_600_ ∼0.6) and diluted into fresh media. The *A*_600_ was recorded every 20 min in an Epoch2 microplate reader (BioTek) to determine the doubling times. For growth assays in the presence of translation inhibitors, the following concentrations were used: 500 μg/ml paromomycin, 15 μg/ml hygromycin B, 10 μg/ml anisomycin, and 500 μg/ml homoharringtonine. For spot growth assays, cells were grown to saturation and serially diluted in sterile water, spotted on plates, and grown at 30 °C or 37 °C.

### Western blot analysis

*hit1*Δ cells transformed with HA-Hit1 expression plasmids (see Table S3) were grown to mid-log phase in minimal media lacking histidine. An equivalent of 10 ml cells at *A*_600_ 0.6 was harvested, washed, and lysed in 1 ml SUMEB buffer (1% SDS, 8 M urea, 10 mM MOPS pH 6.8, 10 mM EDTA, 0.01% bromophenol blue) with glass beads. Lysates were analyzed by SDS-PAGE on a Mini-PROTEAN TGX Stain-Free precast gel (Bio-Rad), followed by Western blotting. A high-affinity anti-HA antibody (Roche) was used for the detection of HA-tagged Hit1 and an anti-PGK1 antibody (Thermo Fisher Scientific) was used to detect the control protein. Bands were quantified using Image Lab (Bio-Rad).

### Northern blot analysis

Total cell RNA was isolated from cells grown to *A*_600_ ∼0.6, in biological triplicates, using the hot phenol method. For analysis of rRNA processing defects, RNAs were separated on a 1% agarose/formaldehyde gel and passively transferred to a Hi-bond nylon membrane. Membranes were probed using oligos listed in Table S2, and bands were quantified in Image Lab (Bio-Rad).

### RT-qPCR

Reverse transcription was performed with 1 μg of total cell RNA extracted using the hot phenol method. RNA was treated with DNase I (NEB) according to the manufacturer’s instructions and reverse transcribed with 50 ng random hexamers using 200 U SuperScript III reverse transcriptase (Invitrogen) in a 20 uL reaction for 10 min at 25 °C followed by 50 min at 50 °C. qPCR was performed using the oligos described in Table S2 with the Maxima SYBR Green qPCR master mix (Thermo Fisher Scientific) on a BioRad CFX96 instrument. RNA levels were calculated using the 2^-ΔΔCt^ method and were normalized to Alg9 mRNA and plotted relative to wildtype BY4741.

### Sucrose density gradient isolation of polysomal RNA

Cells were grown to mid-log phase in YPD and harvested after addition of 0.1 mg/ml cycloheximide. Harvested cells were washed and lysed in ice-cold gradient buffer (200 mM Hepes-KOH pH 7.4, 1 M KOAc pH 7.6, 25 mM Mg(OAc)_2_), supplemented with 0.1 mg/ml cycloheximide, PMSF, pepstatin, E64, and complete protease inhibitor cocktail (Roche). Cleared lysate was applied to 10 to 50% sucrose gradients in gradient buffer and centrifuged for 2 h at 40,000 RPM in an SW41Ti rotor (Beckman Coulter). Gradients were fractionated and scanned by UV 260 nm absorbance. Fractions corresponding to polysomes were pooled together for analysis of their rRNA 2′-O-methylation pattern by RTL-qP and RiboMethSeq.

### Analysis of global 2′-O-methylation levels by reverse transcription

The 2′-O-methylation level of 25S rRNA was assayed at three sites as previously described (67). Briefly, 1 μg total cell RNA extracted using the hot phenol method was treated with DNase I (NEB) according to the manufacturer’s instructions, then reverse transcribed with 50 ng random hexamers in a high dNTP concentration (20 mM) or low dNTP concentration (0.1 mM) using 200 U SuperScript III reverse transcriptase (Invitrogen) in a 20 uL reaction for 50 min at 50 °C. Samples were treated with RNase H, and cDNAs were analyzed by qPCR using the QuantiTect SYBR Green enzyme and dye mixture (Qiagen) with the oligos listed in Table S2. The following thermocycler setup was used in a StepOnePlus instrument (Applied Biosystems): 15 m at 95 °C followed by 40 cycles of 15 s at 94 °C, 30 s at 55 °C, and 30 s at 72 °C. The quantification cycle delay for each region was calculated as follows: ΔC_q_ = low dNTP C_q_ – high dNTP C_q_. For each region, this value was normalized to the threshold cycle delay of the unmethylated 25S region (Region 1). For analysis of rRNA 2′-O-methylation levels of polyribosomes, the protocol above was used with 700 ng RNA extracted from polysome fractions. Experiments were performed for samples from three biological replicates.

### RiboMethSeq analysis of rRNA 2′-O-methylation pattern

For RiboMethSeq, 150 ng polysomal RNA was fragmented under denaturing conditions using an alkaline buffer (pH 9.4) to obtain an average size of 20 to 40 nt. Fragments were end-repaired and ligated to adapters using NEBNext Small RNA kit for Illumina. Sequencing was performed on Illumina HiSeq1000. Reads were mapped to the yeast rDNA sequences, and the RMS score (fraction methylated) was calculated as MethScore (for ±2 nt) (88), equivalent to “ScoreC” (30).

### Analysis of global translation by HPG incorporation

Translation of newly synthesized peptides was measured with the Click-iT HPG Alexa Fluor 488 Protein Synthesis Assay Kit (Thermo Fisher Scientific) according to the manufacturer’s instructions. BY7471, *hit1-C11F*, or *hit1-S29L* cells transformed with pRS411 plasmid were grown in minimal media lacking methionine to late log phase. HPG was added to 10 ml cultures to a final concentration of 50 μM, and cells were incubated for 30 min at 37 °C. Negative controls received cycloheximide at a final concentration of 0.1 mg/ml before the addition of HPG. Flow Cytometry measurements were done at the Emory Flow Cytometry Core. Cells were fixed, permeabilized, and subjected to click chemistry following the manufacturer’s instructions. Cells were passed through a 0.37 μm Nitex mesh before flow cytometry analysis of fluorescence using the BD FACSymphony A3 Cell Analyzer. The fluorescence intensity of 50,000 to 100,000 cells was determined.

### Dual-luciferase assays for translation fidelity

Cells were grown to *A*_600_ ∼0.6 in Ura^-^ or Leu^-^ synthetic glucose liquid media. One milliliter of cells were pelleted, washed, and stored at −80 °C. Luciferase activities were measured using the Dual-Luciferase Reporter Assay kit (Promega). Cell pellets were resuspended in 100 μl 1 X Passive Lysis Buffer and incubated for 10 min. Thirty microliter of LARII was mixed with 10 μl of lysate in clear bottom 96W Microplates (Costar), and Firefly luciferase activity was measured. Thirty microliter of Stop and Glo solution was added, and Renilla luciferase activity was measured. Measurements were performed using a Synergy Microplate reader (BioTek). For each replicate, the Firefly luciferase signal was normalized to the Renilla luciferase signal. For each strain, Firefly/Renilla ratio was normalized to the average Firefly/Renilla ratio of replicates containing a control plasmid.

## Data availability

Data from RiboMethSeq analysis are deposited with European Nucleotide Archive under accession number PRJEB48888. All other materials are available upon request to the Lead Contact, Homa Ghalei (hghalei@emory.edu).

## Supporting information

This article contains supporting information (46, 47, 48, 49, 66).

## Conflict of interest

The authors declare that they have no conflicts of interest with the contents of this article.
