# Supplementary material for: Studies of mutations of assembly factor Hit1 in budding yeast suggest translation defects as the molecular basis for PEHO syndrome
Source: J Biol Chem. 2022 Jul 14;298(9):102261. doi: 10.1016/j.jbc.2022.102261 (PMC9418376; doi:10.1016/j.jbc.2022.102261)
Supplement: Supplementary Information [file mmc1.docx]

**SUPPLEMENTARY INFORMATION**

**Supplementary Figures: S1-S3**

**
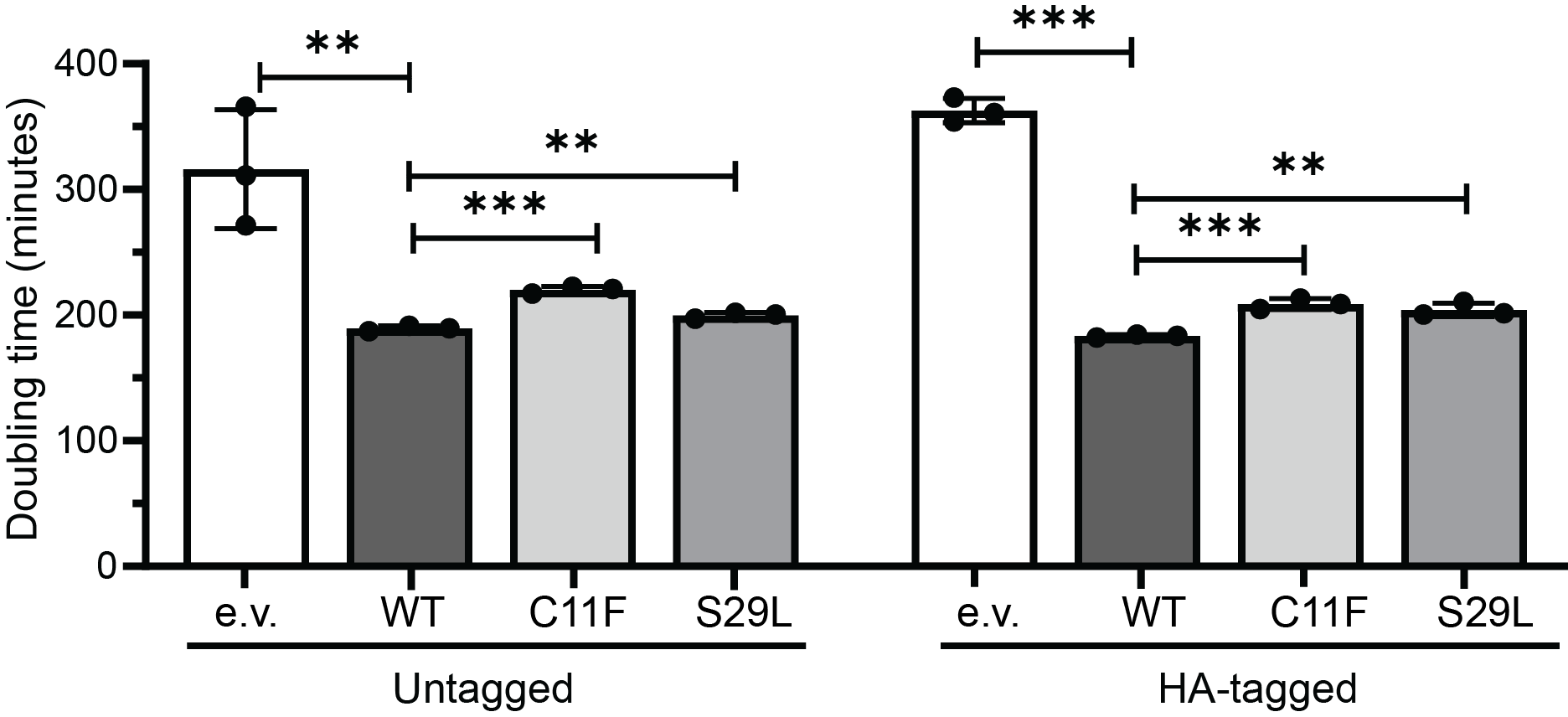
**

**Figure S1. HA-tagged *HIT1* mutant yeast have the same slow-growth phenotype as untagged *HIT1* mutant yeast.** Doubling times of *hit1∆* yeast expressing 3xHA-tagged or untagged *Hit1*, *hit1-C11F*, *hit1-S29L*, or an empty vector is shown for cells grown in minimal media at 37ºC. Bars represent the mean and standard deviation of 3 biological replicates. Significance was determined using an unpaired t-test. **: P<0.01; ***: P<.001.


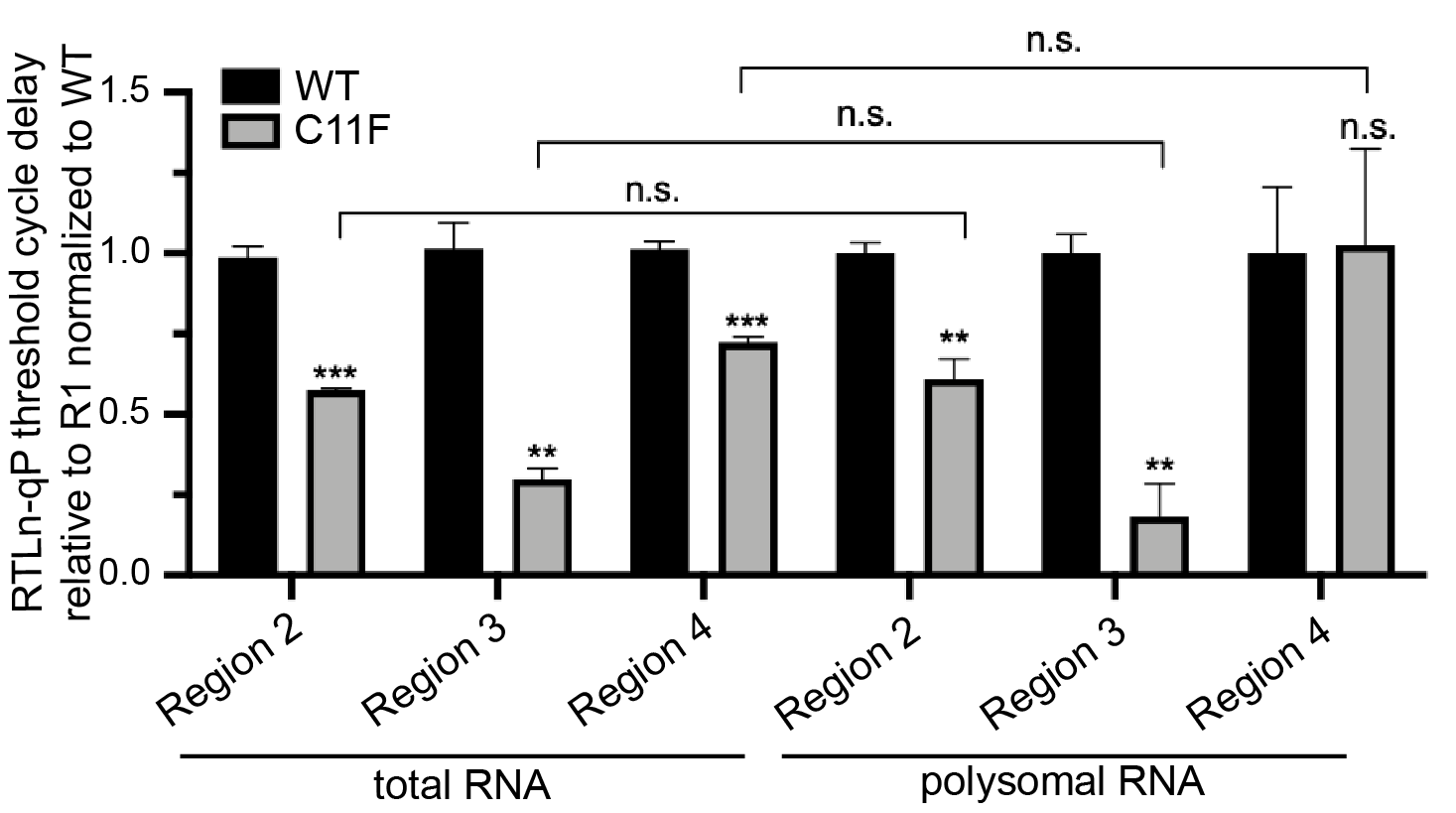


**Figure S2. Ribosomal RNA 2’-O-methylation is not significantly altered between the total and polysomal RNA pools.** Total RNA or polysomal RNA from wild-type control and *hit1-C11F* cells were extracted, and three regions of 25S rRNA were probed for their 2’-O-methylation levels by reverse transcription at low dNTP concentration followed by qPCR in biological triplicate. Bars represent the mean and SD of 2-3 biological replicates. Significance is shown relative to wild-type control. n.s.: not significant; **: p<0.01; ***: p<0.001.


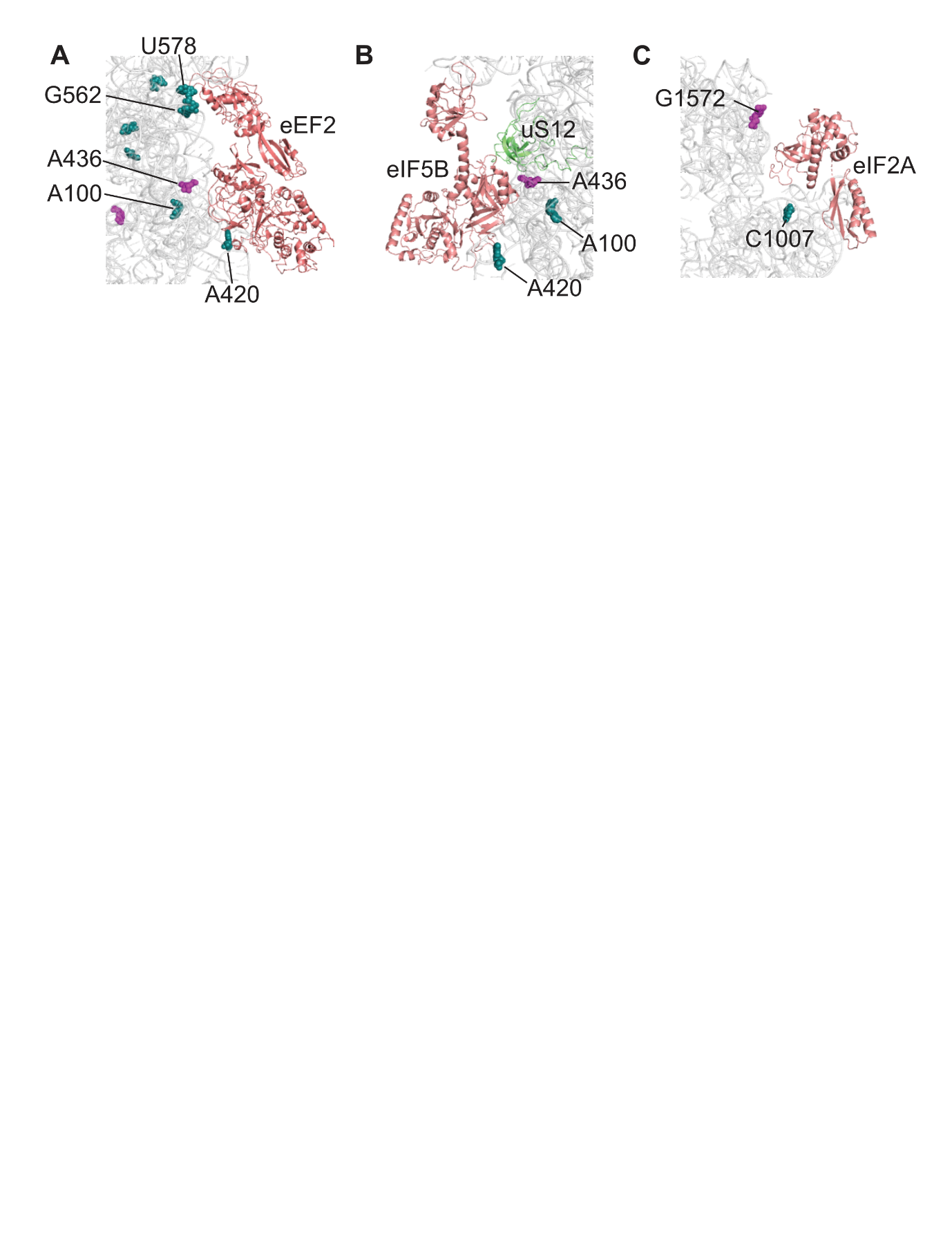


**Figure S3.** Views of the small ribosomal subunit showing variable and hypo 2’-O-methylated sites near eEF2 (**A**), eIF5B (**B**), eIF2A (**C**) binding sites. Translation factors located near hypo 2’-O-methylated sites are colored in salmon red (PDB IDs: 6GQV, 4V8Z, 6FYX), and the ribosomal protein uS12, located near the hypo 2’-O-methylated site A436, is colored in lime green.

**Supplementary Tables: S1-S3**

**Table S1**: **Yeast strains used in this work**

| **Strain name** | **Description** | **Background** | **Reference** |
| --- | --- | --- | --- |
| yHG000 | Wild-type | BY4741 | GE Dharmacon |
| yHG007 | *hit1Δ* | BY4741 | Euroscarf |
| yHG473 | *hit1-C11F* | BY4741 | This work |
| yHG545 | *hit1-S29L* | BY4741 | This work |

**Table S2**: **Oligonucleotides used in this work**

| **Use** | **Oligo** | **Sequence (5’ to 3’)** |
| --- | --- | --- |
| Northern probes | MRP | AATAGAGGTACCAGGTCAAGAAGC |
|  | P1 (between A2 and A3) | TGTTACCTCTGGGCCC |
|  | P2 (ITS2) | GGCCAGCAATTTCAAGTTA |
|  | P3 (between D and A2) | GCTCTCATGCTCTTGCC |
|  | P4 (25S) | GCCCGTTCCCTTGGCTGTG |
|  | P5 (18S) | CATGGCTTAATCTTTGAGAC |
| Site-directed mutagenesis | *HIT1-C11F* | GCAGTTAAATGTGGCATATtTCGCGGAGTTGATGGTAAAT |
|  | *HIT1-S29L* | GTGGCGTACGATATTGCTtgTTGAAATGCTATAAAGATGC |
| Cloning | *HIT1- forward* | CGATAGGATCCATGGTATCTAGTGCAGTTAAATGTGGC |
|  | *HIT1- reverse* | CGATACTCGAGTTATTTCTTCACCGCATTTAATTTATC |
|  | *HIT1 400bp upstream- forward* | GCAGCGGATCCGAGGAGAACTTCTAGTATATCTACATACC |
|  | *HIT1- 300bp downstream- reverse* | GAGCCTCGAGGGAACTTTCCCTAATCATATGTAAATACAT |
| CRISPR | *Hit1 gRNA mutagenesis* | CGGGTGGCGAATGGGACTTTGTTAAATGTGGCATATGTCGGTTTTAGAGCTAGAAATAGC |
|  | *Hit1 repair oligo amplification- forward* | GAGGAGAACTTCTAGTATATCTACATACC |
|  | *Hit1 repair oligo amplification- rev* | GGAACTTTCCCTAATCATATGTAAATACAT |
| qPCR | 25S R1 forward | ACCGGGATTGCCTTAGTAACG |
|  | 25S R1 reverse | TCCTCAGTCCCAGCTGGCAG |
|  | 25S R2 forward | GACGTAAGTCAAGGATGCTGGC |
|  | 25S R2 reverse | GCACCTTAACTCTACGTTCGGTTC |
|  | 25S R3 forward | CTTAGAACTGGTACGGACAAGGG |
|  | 25S R3 reverse | CCCACTTATTCTACACCCTCTATGTCTC |
|  | 25S R4 forward | GGCTGATCCGGGTTGAAGAC |
|  | 25S R4 reverse | CTCGTACTAAGTTCAATTACTATTGCGG |
|  | snR51-F | GATGATTTTTTTATATTCACACTGTACTAG |
|  | snR51-R | CAGAGTCATTAAATCAGTTATTTTTTATTTTG |
|  | snR52-F | TGAATGACATTAGCGTGAACAA |
|  | snR52-R | TTCAGAAGGAAGGCAACATAAG |
|  | U14-F | TCACGGTGATGAAAGACTGG |
|  | U14-R | AAGAGCGGTCACCGAGAGTA |
|  | U24-F | TCAAATGATGTAATAACATATTTGCTACTTC |
|  | U24-R | TTCATCAGAGATCTTGGTGATAATTGG |
|  | snR8-F | CCGTAGGTGATCAGAGGGG |
|  | snR8-R | GGGCCAATGGGAGACAC |
|  | snR46-F | GCGAGGCCTAAATTAACGATC |
|  | snR46-R | GGATGCATGGAAATAGCACAAC |
|  | Alg9-F | TGTCACGGATAGTGGCTTTG |
|  | Alg9-R | TACCATTCACGTCCCGTACA |
|  | 18S-F | GTGCATGGCCGTTCTTAGTTG |
|  | 18S-R | AGGTTAAGGTCTCGTTCGTTATCG |
|  | 25S-F | CCGGGATTGCCTTAGTAACG |
|  | 25S-R | GGCACCGAAGGTACCAGATTT |

**Table S3**: **Plasmids used in this work**

| **Plasmid** | **Description** | **Backbone** | **Reference** |
| --- | --- | --- | --- |
| Addgene #60847 | pCas | 2-micron/pUC | (1) |
| pHG3199 | pCas-Hit1 | 2-micron/pUC | This work |
| pHG384 | *TEF::3xHA-Hit1* | pRS413 | This work |
| pHG3198 | *TEF::3xHA-hit1-C11F* | pRS413 | This work |
| pHG3223 | *TEF::3xHA-hit1-S29L* | pRS413 | This work |
| pHG3268 | *TEF::Hit1* | pRS413 | This work |
| pHG3269 | *TEF::hit1-C11F* | pRS413 | This work |
| pHG3270 | *TEF::hit1-S29L* | pRS413 | This work |
| pDB722 | PGK-Readthrough | pYEplac195 | (2) |
| pDB723 | PGK- Stop | pYEplac195 | (2) |
| pDB868 | PGK- Miscoding Firefly H245R | pYEplac195 | (3) |
|  | pRaugFuug ADH/GPD start | pRS416 | (4) |
|  | pRaugFaug ADH/GPD start | pRS416 | (4) |
| pSRT209 | dicistronic reporter with an wild-type CrPV IGR IRES and a ∆AUG for the luciferase codon that reduces background from cryptic promoters | pRS425 | (5) |
| pSRT210 | identical to pSRT209 except for the 2 nucleotide change in the IRES to disrupt PKI | pRS425 | (5) |

**References:**

1. Ryan, O. W., Poddar, S., and Cate, J. H. (2016) CRISPR-Cas9 Genome Engineering in Saccharomyces cerevisiae Cells. *Cold Spring Harb Protoc* **2016**

2. Keeling, K. M., Lanier, J., Du, M., Salas-Marco, J., Gao, L., Kaenjak-Angeletti, A., and Bedwell, D. M. (2004) Leaky termination at premature stop codons antagonizes nonsense-mediated mRNA decay in S. cerevisiae. *RNA* **10**, 691-703

3. Salas-Marco, J., and Bedwell, D. M. (2005) Discrimination between defects in elongation fidelity and termination efficiency provides mechanistic insights into translational readthrough. *J Mol Biol* **348**, 801-815

4. Cheung, Y. N., Maag, D., Mitchell, S. F., Fekete, C. A., Algire, M. A., Takacs, J. E., Shirokikh, N., Pestova, T., Lorsch, J. R., and Hinnebusch, A. G. (2007) Dissociation of eIF1 from the 40S ribosomal subunit is a key step in start codon selection in vivo. *Genes Dev* **21**, 1217-1230

5. Landry, D. M., Hertz, M. I., and Thompson, S. R. (2009) RPS25 is essential for translation initiation by the Dicistroviridae and hepatitis C viral IRESs. *Genes Dev* **23**, 2753-2764
